# Stacking Effects on Anthraquinone/DNA Charge-Transfer Electronically Excited States

**DOI:** 10.3390/molecules25245927

**Published:** 2020-12-15

**Authors:** Gustavo Cárdenas, Juan J. Nogueira

**Affiliations:** 1 Chemistry Department, Universidad Autónoma de Madrid, Calle Francisco Tomás y Valiente, 7, 28049 Madrid, Spain; gustavo.cardenas@uam.es; 2 IADCHEM, Institute for Advanced Research in Chemistry, Universidad Autónoma de Madrid, Calle Francisco Tomás y Valiente, 7, 28049 Madrid, Spain

**Keywords:** photodynamic therapy, charge transfer, electronically excited states, anthraquinone, DNA, molecular dynamics, TD-DFT, transition-density analysis

## Abstract

The design of more efficient photosensitizers is a matter of great importance in the field of cancer treatment by means of photodynamic therapy. One of the main processes involved in the activation of apoptosis in cancer cells is the oxidative stress on DNA once a photosensitizer is excited by light. As a consequence, it is very relevant to investigate in detail the binding modes of the chromophore with DNA, and the nature of the electronically excited states that participate in the induction of DNA damage, for example, charge-transfer states. In this work, we investigate the electronic structure of the anthraquinone photosensitizer intercalated into a double-stranded poly(dG-dC) decamer model of DNA. First, the different geometric configurations are analyzed by means of classical molecular dynamics simulations. Then, the excited states for the most relevant poses of anthraquinone inside the binding pocket are computed by an electrostatic-embedding quantum mechanics/molecular mechanics approach, where anthraquinone and one of the nearby guanine residues are described quantum mechanically to take into account intermolecular charge-transfer states. The excited states are characterized as monomer, exciton, excimer, and charge-transfer states based on the analysis of the transition density matrix, and each of these contributions to the total density of states and absorption spectrum is discussed in terms of the stacking interactions. These results are relevant as they represent the footing for future studies on the reactivity of anthraquinone derivatives with DNA and give insights on possible geometrical configurations that potentially favor the oxidative stress of DNA.

## 1. Introduction

Photodynamic therapy (PDT) is nowadays a widely-employed technique to treat different types of cancer as well as some infectious diseases [1,2,3,4]. The reason for its widespread usage stems from the fact of being a non-invasive technique which allows for the induction of cell-death through apoptosis on specific target cells, e.g., those present on tumor tissues [5,6]. PDT involves the usage of two main components, namely a photosensitizer (PS) compound and irradiation of light at a specific wavelength to promote the excitation of the PS [7]. The mechanism of PDT apoptosis induction depends on the nature of the PS and on the tissue where the PS accumulates [8,9]. In regard with the mechanism of action of the PS, it is well recognized that after its photoexcitation and population of the triplet-state manifold, it promotes oxidative stress on the surrounding molecules either via electron transfer directly to these molecules (e.g., DNA bases) to produce free radicals (type I mechanism), or through energy transfer to molecular oxygen, which generates singlet oxygen that can cause damage to nearby biomolecules (type II mechanism). Many tumors develop hypoxia conditions [10], where the low flux of oxygen strongly limits the use of PSs that operate via type II reactions. As a consequence, there is a growing interest in the development of PSs that are able to induce damage in the absence of oxygen, such as transition metal complexes [11,12], organic-based compounds [13,14], and nanoparticles [15,16].

It has been evidenced that, depending on its nature, the PS can accumulate in specific cell components such as lysosomes, plasma membrane, mitochondria, the Golgi apparatus or the endoplasmic reticulum [9]. Among these, mitochondria result to be an ideal target since they release pro-apoptopic factors to the cytosol following mitochondrial dysfunction due to mutations in the mitochondrial genome and rupture of the mitochondrial membrane [17,18,19]. In addition, photosensitizers can also cause DNA damage and their interaction with the DNA double strand has been of great interest since this allows for understanding the apoptotic way induced by DNA lesion. It has been established that the mechanism of oxidative stress of DNA following the excitation of the PS depends on the binding mode of the latter, as the electronic structure of the PS can be modified in a specific manner, depending on the surrounding environment [20,21,22,23]. There are three different binding modes in which the PS can bind in a noncovalent manner with DNA [24,25], namely, electrostatic binding, groove binding, and intercalative binding. Although the same PS could bind to DNA through more than one interaction mode [26,27,28,29], it has been evidenced that the preference for a binding mode over the others can be induced by suitably modifying the substituents of the PS [30,31] so that the PS can be tailored to induce oxidative stress on DNA in a specific manner.

Several families of compounds have been studied and tested on PDT, such as cyanines, phenotiazinium dyes, porphirins, phenantridinium dyes, anthraquinones, and acridines, among others [24,32]. Of these, anthraquinone derivatives have shown promising phototoxic activity in vitro on human carcinogenic tissues, especially on breast cancer cells [33,34,35]. Several anthraquinone derivatives are known to interact with DNA through intercalation between two consecutive base pairs [36,37,38,39], and the cleavage efficiency of DNA is strongly dependent on the substituents present on the anthraquinone scaffold [36]. Therefore, the investigation of the binding modes of these photosensitizers and their influence on the nature of the electronically excited states that lead to photoreactions with DNA is of utmost importance for the design of novel anthraquinone-based phototerapeutic drugs. Molecular modeling has proven itself to be a valuable tool in this regard [40]. For example, molecular dynamics (MD) simulations have been employed to study the binding modes and determine the corresponding binding free energies of some representatives of the above mentioned photosensitizer families [41,42,43,44]. Moreover, MD in conjunction with hybrid quantum mechanics/molecular mechanics (QM/MM) approaches have been applied to unravel in a comprehensive manner the binding modes with DNA and the nature of the excited states that give rise to photochemoterapeutic reactivity of organic photosensitizers, such as acetophenone [45], palmatine [46], methylene blue [23,47], Nile red and Nile blue [21], and chelerythrine [48]. Although several MD studies have been performed to unveil the energetics of the noncovalent binding process of anthraquinone derivatives with DNA, in particular the intercalation binding mode [41,44,48,49], to our knowledge a detailed study considering the effect of the DNA surrounding environment on the electronic structure of an anthraquinone derivative has not been performed to this date. Furthermore, in the optics of tailoring more efficient anthraquinone PS derivatives, in particular molecules presenting moieties that favor specific conformations that enhance charge transfer between the PS and the DNA strand, a good place to start would be to consider the pristine anthraquinone (AQ) molecule and to analyze the nature of its electronic structure right after excitation. It is important to note that AQ is not water soluble and, thus, it is unlikely to be employed in PDT mechanisms in biological environments. However, a detailed analysis of its electronically excited states when it is interacting with DNA is important to carry out future comparisons with functionalized water-soluble anthraquinone derivatives with potentially efficient PDT mechanisms.

Herein we present the study of the different rotational poses assumed by the AQ molecule when intercalated between two base pairs of a solvated double-stranded poly(dG-dC) polynucleotide model, and the influence of these different poses on the electronically excited states of AQ at the Franck-Condon region. We have chosen poly(dG-dC) since guanine presents the lowest oxidation potential of all four DNA nucleobases [50], and it has been evidenced that when employing different AQ derivatives, DNA oxidative damage occurs by photoinduced electron transfer from a guanine moiety of DNA to the photoexcited PS [51,52]. The exploration of the ground-state potential-energy surface of the solvated AQ-DNA complex is performed by means of classical MD sampling. Four different conformational minima are identified when analyzing the relative orientation between AQ and one of the two guanine–cytosine flanking base pairs. Then, we investigate the nature of the excited states of AQ depending on its relative orientation with respect to the guanine–cytosine base pair by means of a hybrid electrostatic-embedding QM/MM scheme, in which AQ and a guanine molecule are part of the QM region, whereas the surrounding environment is considered by a MM force field. With this approach and by performing a suitable wavefunction analysis [53], we are able to characterize the different classes of excited states of the system, including those with a high electron-transfer character from the guanine moiety to the AQ molecule, which are relevant in the PDT mechanism of the PS.

## 2. Results and Discussion

### 2.1. Sampling the Stacking Binding Pocket

Molecular PSs formed by fused-ring aromatic moieties are known to non-covalently bind to DNA strands as intercalators between neighboring base pairs, where the PS/DNA complex is stabilized by stacking interactions between the aromatic rings of the drug and the nucleobases [24,25]. This is the case of anthraquinone derivatives, whose DNA intercalative binding have been extensively investigated [36,38,54,55]. Despite the presence of strong staking interactions, both the PS and the nucleobases can undergo large molecular motions, inducing important conformational changes in the system. For example, the PS can rotate inside the intercalative pocket of DNA, as was found for methylene blue by MD simulations [43] and spectroscopic measurements [56], or one or two nucleobases can be ejected and replaced by the intercalator, as it was observed for benzophenone by MD simulations [42,57]. Therefore, vibrational sampling must be considered in the theoretical model when investigating the photophysics of the PS, since different spatial configurations of the chromophore and the environment can present different electronic properties [58].

**Figure 1:**
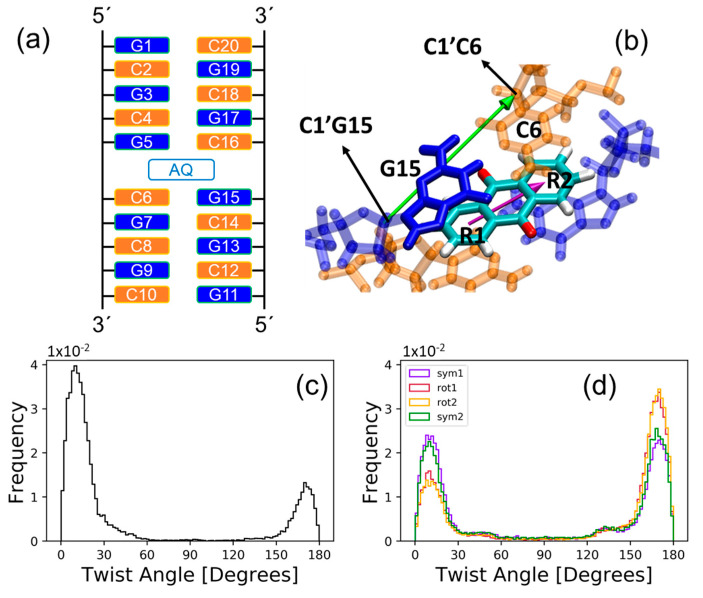
(**a**) Schematic representation of anthraquinone (AQ) intercalated into the d(GCGCGCGCGC) decamer sequence between the G5-C16 and C6-G15 base pairs; (**b**) representation of the long axis of AQ (magenta) and long axis of the G15-C6 base pair (green) used to compute the twist angle; (**c**) probability distribution of the twist angle for the initial 200 ns MD simulation; (**d**) probability distribution of the twist angle for the four 200 ns MD simulations which were initialized by four snapshots selected from the four configuration regions (symmetric 1 and 2 and rotated 1 and 2) of the initial simulation. Color code: Guanine nucleotide residues are represented in blue, cytosine nucleotides in orange, and the C, O, and H atoms of AQ in cyan, red and white, respectively.

The AQ molecule was introduced between the fifth and sixth guanine–cytosine base pairs (G5-C16 and C6-G15) in the double-stranded d(GCGCGCGCGC) decamer as shown in Figure 1a. Then, a classical MD simulation was evolved for 200 ns. We observe large rotational motions of AQ inside the pocket, which can be monitored by computing the twist angle formed by the long axis of AQ and the long axis of the G15-C6 base pair. The former is defined as the vector that connects the centers of mass of the two outer benzene rings of anthraquinone (R1 and R2), and the latter is defined as the vector that connects the C1′ atoms of the sugars of each nucleoside in the G15-C6 base pair, as is shown in Figure 1b. The probability distribution of the twist angle, plotted in Figure 1c, presents 2 intense maxima at the regions of 0–30° and 150–180°, which correspond to spatial configurations with strong stacking interactions between AQ and the flanking base pairs. We will refer to these two PS orientations as symmetric configurations 1 and 2, respectively. These two distribution maxima extend over the regions of 30–60° and 120–150° with much less intensity, where the chromophore rotated around the axis normal to its aromatic plane and partially broke the stacking interactions with the nucleobases. These two spatial orientations will be named rotated configurations 1 and 2, respectively. Therefore, the twist angle distribution indicates that the PS visits preferentially four regions of the potential-energy surface: two symmetric and two rotated configurations. In order to determine whether these four configurations are stable or whether they are consequence of a bad equilibration of the system along the simulation, four additional MD trajectories of 200 ns each were performed. The initial conditions for these new simulations are taken from four different snapshots selected from the symmetric and rotated configurations of the original simulation. The twist angle probability distribution for the four additional simulations are shown in Figure 1d. As can be seen, the same two symmetric and two rotated configurations are clearly identified. Thus, one can conclude that they are stable regions of the potential-energy surface that must be considered in the subsequent electronically excited-state calculations.

**Figure 2:**
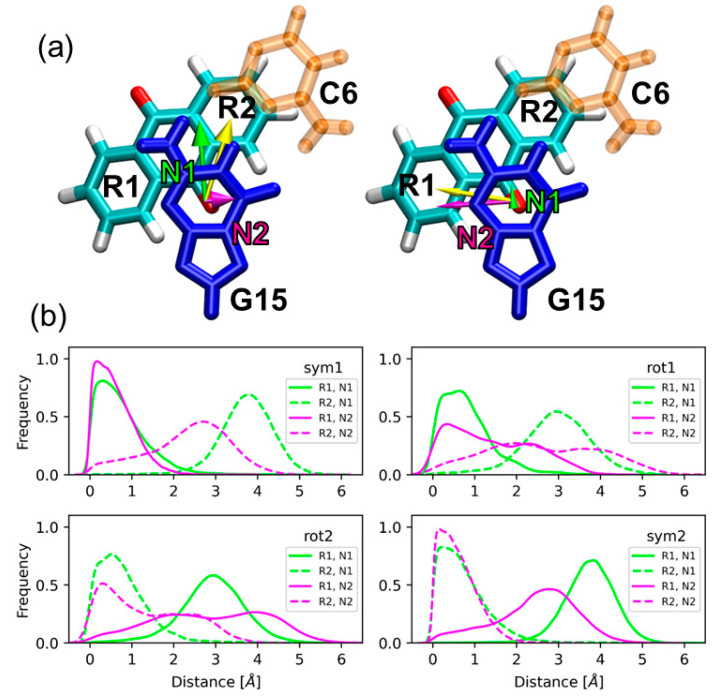
(**a**) Shift (N1, green) and slide (N2, magenta) distances between guanine G15 and the rings R1 and R2 of AQ employed in the analysis of the stacking interactions. These distances are computed as the separation between the center of mass of the six-membered ring of guanine and the center of mass of each benzene ring R1 and R2 of AQ (yellow vector) projected on the guanine plane, and along the base-pair direction (N1) and the direction perpendicular to it (N2); (**b**) probability distributions of the shift and slide distances for the two symmetric and the two rotated configurations. Color code: Guanine in blue, cytosine in orange, and the C, O, and H atoms of AQ in cyan, red, and white, respectively.

The twist angle defined above shows that the stacking interactions between AQ and the flanking bases are stronger for the symmetric configurations than for the rotated ones. However, a more rigorous geometrical analysis can be performed to characterize the stacking interactions for the symmetric and rotated configurations, and the influence of stacking on the electronically excited states of the system. As will be discussed later, the excitation energies of the system were computed by an electrostatic-embedding QM/MM scheme, where AQ and the nucleobase G15 (see Figure 1a,b) were included in the QM region. This partition of the system allows the investigation of delocalized excitations, where both the chromophore and the nucleobase participate, and of the effect of the stacking interactions on those excitations. Therefore, the stacking interactions present in the symmetric and rotated configurations have been characterized in terms of the relative orientation between AQ and the nucleobase G15. Specifically, two intermolecular coordinates were defined: The shift and slide distances represented in Figure 2a. The shift distances (N1) are computed as the separation between the center of mass of the six-membered ring of guanine and the center of mass of each of the benzene rings of AQ (R1 and R2 in Figure 2a) projected on the plane of guanine and along the base-pair direction. The base-pair direction is defined here as the direction given by the vector that connects the center of mass of guanine and the bisection of the C-C bond opposite to the pyrrole ring of guanine. The slide distance (N2) is defined as the separation between the centers of mass of the six-membered ring of guanine and each of the benzene rings of AQ projected again on the plane of guanine, but along the direction perpendicular to the base-pair direction. This perpendicular direction is given by the vector that connects the center of mass of the six-membered ring of guanine and its carbonyl group, then orthogonalized with respect to N1 via a Gram–Schmidt orthogonalization process. We have calculated two shift distances and two slide distances—with respect to the rings R1 and R2 of AQ—because the visual inspection of the dynamics shows that the AQ ring involved in the stacking interactions with G15 is different depending on the geometric configuration. This can be observed in Figure 2b, which displays the probability distributions of the shift and slide distances for the two symmetric and the two rotated configurations. The orientations symmetric 1 and rotated 1 present shorter slide and shift distances for the ring R1 than for the ring R2, while the opposite is true for the symmetric 2 and rotated 2 orientations. This means that the ring R1 is involved in the stacking interactions in the symmetric 1 and rotated 1 orientations, while the ring R2 is the one that interacts with guanine in the symmetric 2 and rotated 2 orientations. The slide and shift distributions with respect to the ring R1 (R2) for the symmetric 1 orientation are very similar to the distributions for the symmetric 2 orientation with respect to the ring R2 (R1). This indicates that the strength of the stacking interactions is similar for both symmetric configurations. The same holds for the rotated configurations, indicating that the difference in the stacking interactions between the two rotated orientations is not important. However, the rotated configurations exhibit weaker stacking interactions than its symmetric counterparts, as reflected by their widespread slide distributions. The probability distribution for the slide distance (N2) with respect to the ring R1 is extended over larger distances for the rotated 1 orientation than for the symmetric 1 orientation. Similarly, the probability distribution for the slide distance (N2) with respect to the ring R2 is extended over larger distances for the rotated 2 orientation than for the symmetric 2 orientation. The different geometric features observed for the symmetric and rotated configurations, which are related with different stacking scenarios, will be reflected on the electronic properties of the excited states, as will be discussed below.

### 2.2. Electronically Excited States: Delocalization and Charge Transfer

The presence of DNA environment can strongly modify the electronic structure of the excited states of chromophores [23,47,59]. In particular, the formation of stacked complexes can result in collective excitations, where the chromophore and the interacting nucleobases actively participate. The electronically excited states of the solvated AQ/DNA complex studied here were computed by an electrostatic-embedding QM/MM scheme, in which the QM region is composed by AQ and G15 (see Figure 1a) and described by time-dependent density-functional theory with the CAM-B3LYP functional. Then, the excited states are subsequently characterized by electronic-structure descriptors based on the one-particle transition density [60,61,62]. The 10 lowest singlet excited states were computed for 100 snapshots for each of the four geometric configurations discussed above: symmetric 1 and 2 and rotated 1 and 2. Thus, a total of 4000 electronically excited states were computed and characterized. More information can be found in the Materials and Methods section. One of the goals of the present computations and analyses is to characterize the intermolecular charge-transfer states, where electron transfer between the DNA and photosensitizer happens, because those states would potentially lead to DNA damage. Since guanine is the most easily oxidizable nucleobase, we have included only one of the neighboring guanines inside the QM region. The use of larger QM regions would allow the investigation of electronic states that present a larger delocalization along the DNA strand [63]. Therefore, it would be interesting to investigate in future works those delocalized electronic states and how they are influenced by the binding of a photosensitizer.

**Figure 3:**
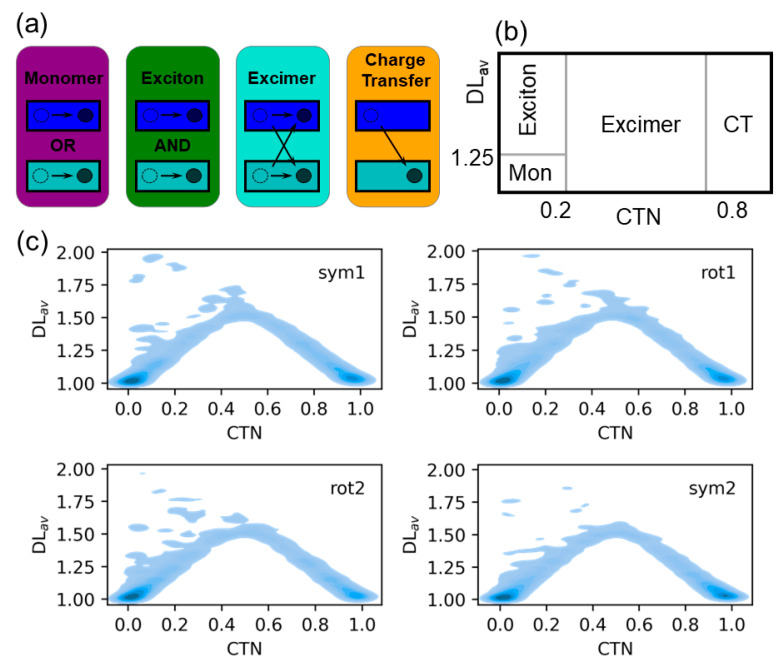
(**a**) Representation of the excited states formed in two fragments, e.g., AQ and guanine, represented by rectangles. Empty and filled circles represent the electron-holes and excited electrons, respectively. The thresholds for the average delocalization length (DL_av_) and charge-transfer number (CTN) descriptors to classify the excited states are given; (**b**) Different areas of the 2-dimensional CTN/DL_av_ distribution where the four types of excited states lie; (**c**) 2-dimensional CTN/DL_av_ probability distributions for the four geometric configurations identified in the dynamics.

The absorption of light by multimeric stacking complexes can lead to the formation of monomer-like excitations, Frenkel excitons, charge-transfer states and excimer states, as represented schematically in Figure 3a for the system investigated here formed by two absorbing fragments: AQ and guanine. Monomer-like excitations occur when the excitation is localized on a single fragment of the system, i.e., on AQ or on guanine. Frenkel excitons are excitations where both the electron-hole and the excited electron are delocalized over the two fragments with no density exchange between fragments. In charge-transfer states the electron-hole and the excited electron are located on different fragments, i.e., the hole is on AQ and the electron on guanine or vice versa. Finally, excimer-like states are a combination of monomer-like and charge-transfer states. It is important to note that the present analysis is aimed to the Franck-Condon region and, thus, excimer species stabilized in an excited-state potential-energy minimum are not formed. However, we use the term excimer states because a strong mix between charge-transfer and exciton states was observed during excimer formation [60]. These four electronic states can be univocally identified by means of the computation of two electronic descriptors, namely average delocalization length (DL_av_) and charge-transfer number (CTN), which have been previously employed to describe the collective excitations in a polyadenine single strand [64]. DL_av_ is the arithmetic mean between the electron-hole and excited-electron participation ratios and indicates the number of fragments over which the hole and electron are delocalized. For example, pure monomer states and pure exciton states delocalized over AQ and guanine will present a DL_av_ value of 1 and 2, respectively. CTN provides the fraction of excited electron (or hole) density transferred between different fragments. For example, pure Frenkel exciton states and pure charge-transfer states will have CTN values of 0 and 1, respectively. The different electronic states were classified using the following DL_av_ and CTN arbitrary thresholds employed in a previous publication [64]: Electronic states with DL_av_ < 1.25 and CTN < 0.2 are considered monomer-like states; excitons are defined as states with DL_av_ > 1.25 and CTN < 0.2; excimers have DL_av_ > 1.25 and 0.2 ≤ CTN ≤ 0.8; and finally, a state is classified as a charge-transfer state if CTN > 0.8. If the 2-dimensional probability distribution function of DL_av_ and CTN is computed, the four different types of states can be easily visualized on different regions of the distribution contour plot (see Figure 3b).

The CTN/ DL_av_ probability distribution for the 100 snapshots of each of the stacking situations (symmetric 1 and 2 and rotated 1 and 2) is plotted in Figure 3c. The most important contribution for all the stacking configurations comes from monomer excitations, which are concentrated on a small region of the contour plot at DL_av_ = 1 and CTN = 0 and, thus, correspond to pure monomer states. Charge-transfer states represent the second most important contribution. As for monomer states, the distribution of charge-transfer states is well localized on a small area of the plot, but in this case around the values of DL_av_ = 1 and CTN = 1. This is especially true for the symmetric orientations, which present a more intense signal than the rotated orientations. This is not surprising since orbital overlap between AQ and guanine is expected to be stronger for the symmetric configurations, where stacking is stronger, leading to more efficient charge-transfer processes. Excimer states are also important but, contrary to monomer-like and charge-transfer states, they are spread over a large area of the distribution map with wide range of CTNs. Finally, exciton states are almost irrelevant and appear mainly on the boundary with excimer and monomer states, that is, the amount of pure Frenkel exciton states are negligible.

**Figure 4:**
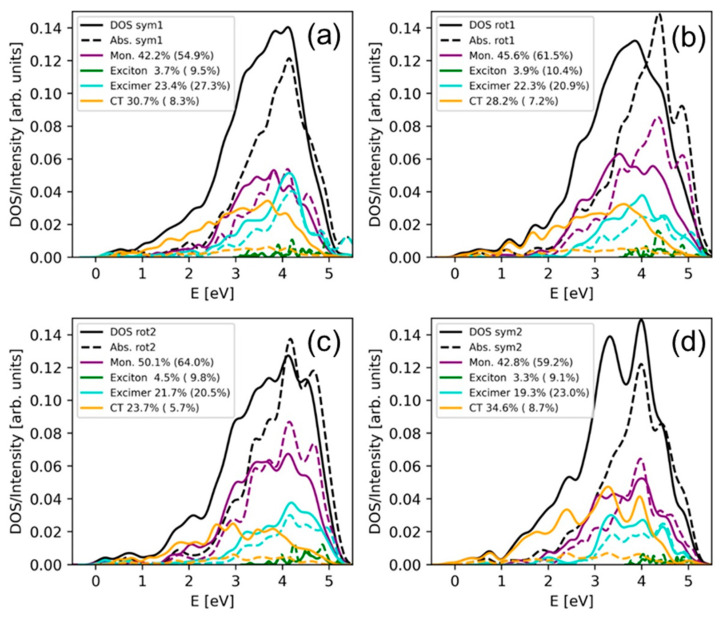
Decomposition of the density of states (solid lines) and absorption spectrum (dashed lines) into monomer, Frenkel exciton, excimer, and charge-transfer states for the (**a**) symmetric 1, (**b**) rotated 1, (**c**) rotated 2 and (**d**) symmetric 2 configurations. The percentage of the different contributions to the density of states (absorption spectrum) are given in the legend.

The exact contribution of each state class to the total density of states composed by the ten lowest singlet states can be seen in Figure 4. Monomer excitations represent around 42% of electronic states for the symmetric configurations, while it increases to 45.6% and 50.1% for the orientations rotated 1 and 2, respectively. The percentage of states with exciton character are lower than 5% independently on the stacking situation. The contribution of charge-transfer states is larger for the configurations symmetric 1 (30.7%) and symmetric 2 (34.6%) than for the configurations rotated 1 (28.2%) and rotated 2 (23.7%) because, as explained above, orbital overlap between fragments is expected to be stronger for the symmetrically stacked orientations. Since excimer states are a mixture of monomer and charge-transfer states, and those behave in an opposite manner with the stacking interactions, there is no correlation between the contribution of excimer states and the stacking scenario. If the intensity of each electronic excitation is considered in the analysis, i.e., if the absorption spectrum (and not the density of states) is decomposed into the different contributions, the situation drastically changes. As Figure 4 displays, the importance of the charge-transfer states is greatly reduced in the absorption spectrum and, consequently, the percentage of the other electronic-state types increases. In other words, most of charge-transfer states are dark because the transition dipole moment from the ground state to charge-transfer states is small due to the relatively large separation between the PS and guanine, which precludes a strong orbital overlap between the interacting chromophores. The presence of excimer states with mixed exciton and charge-transfer character has also been identified in guanine–cytosine duplexes by means of fluorescence measurements and quantum mechanical calculations [65]. Since these states can evolve to charge-transfer states, which are responsible for DNA photodamage, it would be interesting to investigate in future studies the effect of the photosensitizer intercalation on the guanine–cytosine charge-transfer states, especially at low energies, where these states can be easily populated.

**Figure 5:**
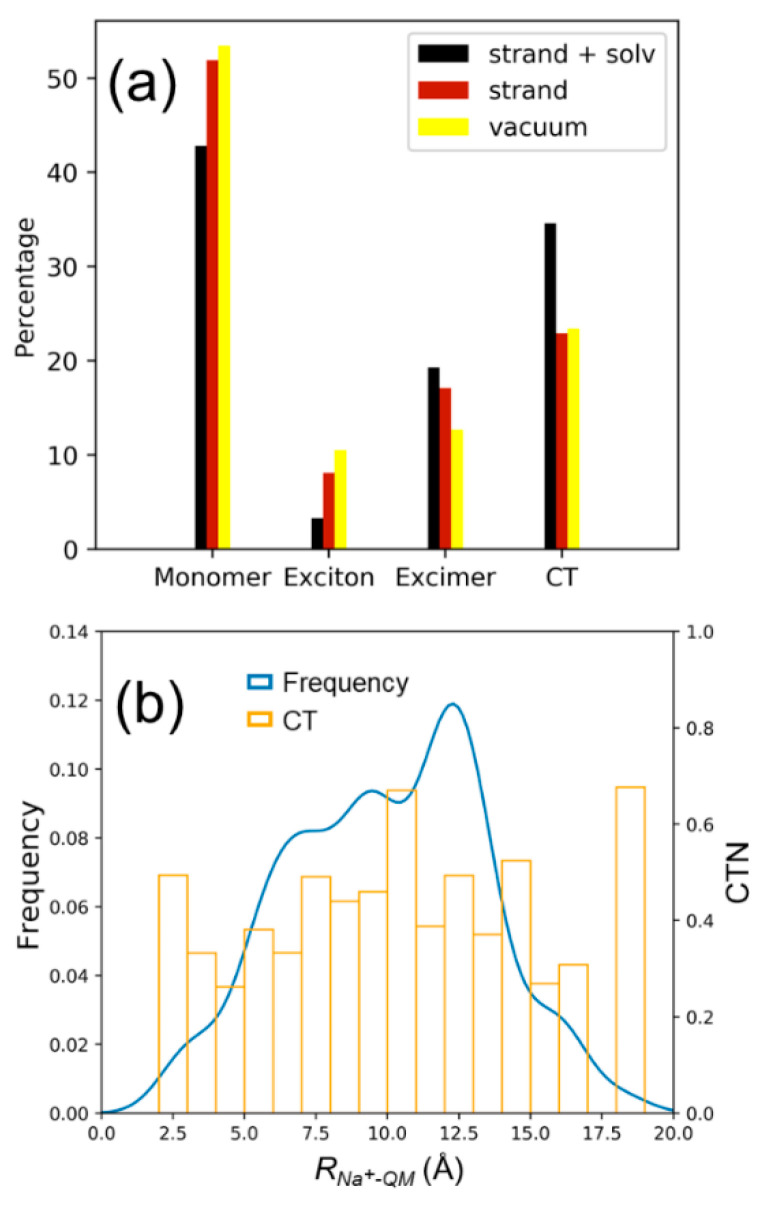
(**a**) Contribution of monomer, exciton, excimer and charge-transfer states to the density of states for the symmetric 2 configuration. The percentages of the full system, the quantum mechanics (QM) region (AQ and G15) embedded in the DNA strand and the QM region in vacuum are shown in black, brown and yellow, respectively. (**b**) Probability distribution of the separation *R_Na_^+^_-QM_* between the center of mass of the QM region and the nearest Na^+^ ion to the QM region (blue) and variation of the CTN with that separation (yellow).

The role of the DNA and solvent environment on the nature of the excited states have also been investigated. Specifically, additional calculations were carried out for the 100 snapshots previously selected from the symmetric 2 configuration. In particular, the solvent molecules and Na^+^ ions have been removed from the model and the excited-state QM/MM calculations have been performed for the AQ-G15 QM region electrostatically embedded in the DNA strand. Then, the classical strand has also been removed and the excitations have been computed only for the QM region in vacuum. In this way, the effect of the classical DNA strand and of the solvent on the nature of the excited states can be easily disentangled. Figure 5a shows the contribution of the different electronic states to the total density of states for the three different models: Full system, QM region embedded in the classical DNA strand, and QM system in vacuum. As can be seen, when the solvent is removed from the model the contribution of charge-transfer states drastically decreases and, consequently, the monomer-like states become relevant. In addition, the percentage of excimer states suffers a slight drop due to the smaller amount of charge-transfer states that are available to be mixed with exciton states to form excimers. When the strand is also removed from the model, the most significant alteration is seen in the excimer states, whose contribution decreases. Thus, the presence of aqueous solvent favors the formation of charge-transfer states—a fact that is not surprising—and the presence of the DNA strand—described as a point-charge electrostatic embedding—favors the formation of excimer states. An additional factor that deserves attention is the role of the Na^+^ ions. In particular, the presence of a positive ion close to the absorbing region of the system could drastically affect the charge-transfer states by stabilizing the excited electron or destabilizing the electron-hole by Coulomb interactions. Figure 5b displays the probability distribution of the separation between the center of mass of the AQ-G15 region and the nearest Na^+^ ion. As can be seen, the position of the nearest ion can adopt a wide range of distances from the QM region from around 1 to 20 Å. However, the CTN of the electronic states does not show a clear trend with the ion position and its value is always around 0.4.

**Figure 6:**
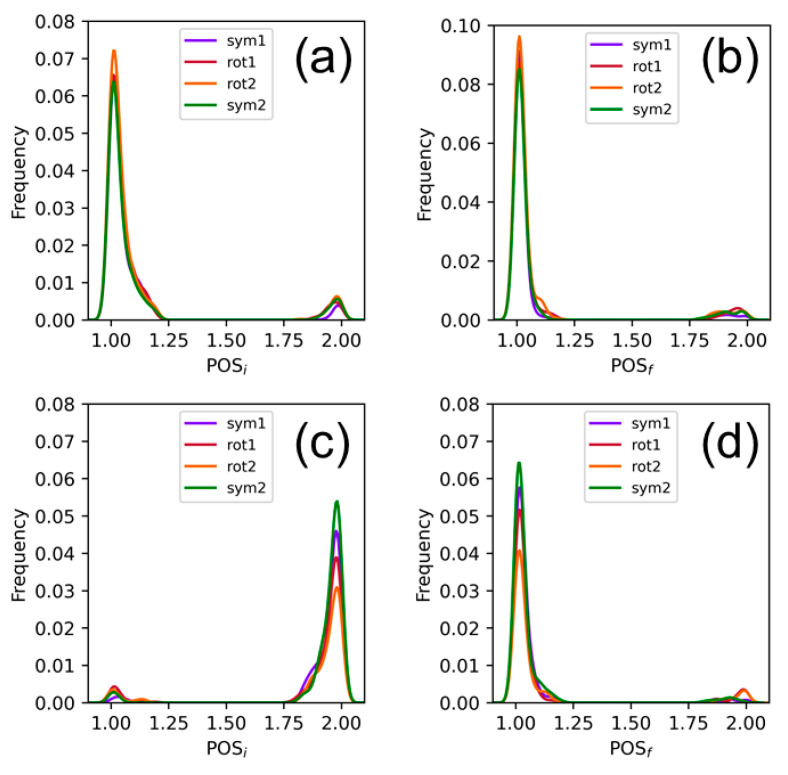
Probability distribution of the position of the hole (POS_i_) and of the position of the excited electron (POS_f_) for (**a**,**b**) the monomer-like states and (**c**,**d**) the charge-transfer states. Positions 1 and 2 correspond to AQ and guanine, respectively.

It is interesting to characterize in more detail the monomer and charge-transfer states. As explained above, monomer states are formed by excitations where both the electron-hole and excited-electron densities are located on the same fragment. To unravel the fragment that is involved in the monomer excitations of Figure 3 and Figure 4, the probability distribution of the position of the electron-hole (POS_i_) and of the position of the excited electron (POS_f_) have been obtained from the transition-density analysis [60], and are plotted in Figure 6a,b. Both distributions peak at fragment 1, which in our case is AQ, and only a small fraction of the distribution appears at fragment 2 (guanine) for the four orientation configurations. This means that most of the monomer-like electronic states that are involved in the density of states are located on the PS. The same analysis performed for the charge-transfer states, plotted in Figure 6c,d, reveals that the electron-hole and excited electron are located at fragments 2 and 1, respectively, independently of the orientation of the chromophore inside the binding pocket. This means that the electron flow in charge-transfer states occurs mainly from guanine to AQ, and only a small percentage of electronically excited states present electron transfer in the opposite direction.

## 3. Materials and Methods

The poly(dG-dC) decamer structure was constructed using the Nucleic Acid Builder (NAB) utility of the Amber18 [66] software. The poly(dG-dC) polynucleotide consisted of a double strand having a 10-base guanine–cytosine sequence in each strand. AQ was non-covalently bound to the polynucleotide model by manually positioning it between the fifth and sixth guanine–cytosine base pairs (G5-C16 and C6-G15 in Figure 1a) of the poly(dG-dC) sequence, to emulate the intercalative binding mode of AQ with DNA. The tleap module of AmberTools19 [66] was used to solvate the AQ-DNA system with a periodic truncated octahedral water solvation box considering a maximum distance of 10 Å from any solute atom to the faces of the box, and a suitable number of Na^+^ ions was introduced to neutralize the phosphate moieties. The polynucleotide was classically described with the OL15 force field [67], whereas the bonding and the Lennard-Jones nonbonding parameters of AQ were taken from the general AMBER force field for organic molecules [68]. Water molecules were described by the TIP3P [69] solvation model and Na^+^ ions by suitable AMBER parameters [70]. The geometry of AQ was optimized at the MP2/6-31G* level of theory using the Gaussian16 [71] software, and restrained electrostatic potential charges (RESP) for AQ were calculated at the HF/6-31G* level of theory using the same software. We performed classical MD simulations using the GPU accelerated pmemd software [72] of the Amber18 package. The entire system was at first minimized for 5000 steps using the steepest descent algorithm, followed by 5000 steps using the conjugate gradient algorithm. Afterwards, the system was gradually heated for 50 ps at constant volume (NVT ensemble), using a timestep of 2 fs, to the temperature of 300 K. During the heating process, positional restraints were used for both AQ and poly(dG-dC), by applying a force constant of 10 kcal/(mol Å^2^), while harmonic restraints having the same force constant were applied on the base pairs at the top and at the bottom of the polynucleotide structure (that is, on the G1-C20 and the C10-G11 base pairs) to conserve the double helix structure. This harmonic potential used an equilibrium distance of 10.5 Å between the centers of mass of the base-pairing nucleotides. After the heating, the entire system was equilibrated at constant pressure (NPT ensemble) by three consecutive 2 ns MD simulations, on which the positional restraints (but not the harmonic restraints on the G1-C20 and the C10-G11 base pairs) were gradually removed to 10, 5, and 0 kcal/(mol Å^2^). Afterwards, a long 200 ns production simulation was performed in the NPT ensemble using Langevin thermostat to keep the temperature constant; the SHAKE [73] algorithm was used along the entire protocol to maintain fixed the bonds involving hydrogen atoms. From the resulting 200 ns trajectory, a snapshot was taken from each one of the two symmetric and from each one of the two rotated configurations identified from the distribution of the twist angle (formed between the long axis of AQ and the axis of the G15-C6 base pair) along the entire trajectory. These four snapshots were used as starting geometries for a 200 ns MD production each, so that, overall, 1000 ns of classical MD simulation were obtained. From the resulting 1000 ns trajectories we sampled 100 geometries from each of the intervals (0,30), (30,60), (120,150), and (150,180) degrees of the twist angle, using the Metropolis Monte Carlo algorithm so that the sampled geometries reproduced the Boltzmann-distributed twist angles along the 1000 ns MD trajectories. For each of these 400 selected geometries an electrostatic embedding hybrid QM/MM calculation was performed using the Amber18 interface with the Gaussian16 software, in which the QM region comprised the AQ molecule plus the G15 guanine nucleobase. In addition, for the 100 geometries of the symmetric 2 configuration, additional calculations were performed for two different models: The QM region electrostatically embedded in the DNA strand and the QM region in vacuum. For the QM calculation, the first 10 singlet excited states were computed at the TD-DFT level with the CAM-B3LYP [74] long-range corrected functional and Dunning’s cc-pVTZ [75] basis set. The CAM-B3LYP functional was used since it has provided an excellent agreement with the experimental absorption spectra of many organic photosensitizers while providing a proper description of the charge-transfer states involved [76,77]. The characterization of the monomer, excimer, Frenkel exciton and charge-transfer states was performed by using the TheoDORE program suite [60,62].

## 4. Conclusions

Anthraquinone derivatives are known to participate in PDT mechanisms through the interaction with DNA strands by, mainly, an intercalative binding mode. After absorption of UV light, the PS is involved in DNA oxidative damage, where electron transfer from guanine nucleobases to the excited PS occurs. Therefore, an efficient photoinduced DNA damage pathway requires the existence of charge-transfer states energetically accessible. In this work, the electronically excited states at the Franck-Condon region of AQ intercalated into a solvated double-stranded d(GCGCGCGCGC) decamer was investigated by means of a combination of classical MD simulations, QM/MM excited-state calculations and one-electron transition-density matrix analysis.

Classical MD simulations evolved for 1 μs showed that the chromophore can adopt four different stable poses inside the intercalative pocket of DNA: two symmetric configurations, where the stacking interactions between AQ and the flanking nucleobases are strong, and two rotated configurations, where the stacking interactions are partially broken. The density of states and the absorption spectrum of the AQ-DNA solvated complex for the four different geometric configurations were computed by means of an electrostatic-embedding QM/MM scheme, where the chromophore and one of the flanking guanine residues were described by TD-DFT in the QM region. For each of the geometric configurations, 100 geometries were considered in the computation of the 10 lowest singlet excited states to take into the account the vibrational sampling of the system. Moreover, the analysis of one-electron transition densities allowed the characterization of the excited states as monomer, Frenkel exciton, excimer and charge-transfer states. The density of states is mainly dominated by monomer-like states located on AQ and charge-transfer states, where an electron is transferred from guanine to the chromophore. Moreover, the charge-transfer states are more relevant for the symmetric configurations, where orbital overlap between AQ and guanine is expected to be more important, than for the rotated configurations. Excimer states are also relatively important in the density of states band, and Frenkel excitons are virtually negligible since they represent less than 5% of the signal. The contribution of charge-transfer states is drastically reduced, while the contribution of the other electronic-state classes increases, when the absorption spectrum is computed, a fact that indicates that the AQ/guanine charge-transfer states are mainly dark.

In conclusion, the binding of AQ to DNA induces the formation of charge-transfer excited states between the PS and guanine. We have shown that these states are energetically accessible at the Franck-Condon region in the singlet manifold. It is very likely that those states are also available in the triplet manifold after intersystem crossing, from where the DNA oxidative damage is initiated. However, further simulations are needed to investigate the efficiency of intersystem crossing when the chromophore is interacting with DNA, and of the charge-transfer process once the system is in the triplet manifold. In addition, theoretical modeling would also be beneficial to rationally functionalize the AQ scaffold and obtain new PSs with improved photophysical properties. The excited-state electronic structure of these functionalized anthraquinone derivatives could be compared with the present calculations for AQ to evaluate whether the functionalization of AQ has led to a modification of the electronic-state features, e.g., the charge-transfer character, that could enhance DNA damage.
